# Differentiating Hepatocellular Carcinoma from Hepatitis C Using Metabolite Profiling

**DOI:** 10.3390/metabo2040701

**Published:** 2012-10-10

**Authors:** Siwei Wei, Yuliana Suryani, G. A. Nagana Gowda, Nicholas Skill, Mary Maluccio, Daniel Raftery

**Affiliations:** 1 Department of Chemistry, Purdue University, 560 Oval Dr., West Lafayette, IN 47907, USA; Email: wei5@purdue.edu (S.W.); ysuryani@purdue.edu (Y.S.); ngowda@uw.edu (G.A.N.G.); 2 Department of Surgery, IU School of Medicine, 550 University Blvd., UH 4601, Indianapolis, IN 46202, USA; Email: nskill@iupui.edu (N.S.); mmaluccio@iupui.edu (M.M.)

**Keywords:** hepatocellular carcinoma, heptatitis C, metabolomics, NMR

## Abstract

Hepatocellular carcinoma (HCC) accounts for most liver cancer cases worldwide. Contraction of the hepatitis C virus (HCV) is considered a major risk factor for liver cancer. In order to identify the risk of cancer, metabolic profiling of serum samples from patients with HCC (n=40) and HCV (n=22) was performed by ^1^H nuclear magnetic resonance spectroscopy. Multivariate statistical analysis showed a distinct separation of the two patient cohorts, indicating a distinct metabolic difference between HCC and HCV patient groups based on signals from lipids and other individual metabolites. Univariate analysis showed that three metabolites (choline, valine and creatinine) were significantly altered in HCC. A PLS-DA model based on these three metabolites showed a sensitivity of 80%, specificity of 71% and an area under the receiver operating curve of 0.83, outperforming the clinical marker alpha-fetoprotein (AFP). The robustness of the model was tested using Monte-Carlo cross validation (MCCV). This study showed that metabolite profiling could provide an alternative approach for HCC screening in HCV patients, many of whom have high risk for developing liver cancer.

## 1. Introduction

Hepatocellular Carcinoma (HCC) is the most common type of liver cancer and the third leading cause of cancer mortality worldwide, especially in China and South East Asia [1]. Although most cases (85%) occur in developing countries, the incidence of HCC in the U.S. has tripled over the past twenty years [2]. The five-year survival rate is very poor, less than 5% [3]. Early diagnosis can give patients an opportunity to receive curative treatments; this then improves outcomes [4]. The current diagnostic methods include cross sectional imaging and biopsy in cases where the imaging does not meet established diagnostic criteria. Once cancer develops in a hepatitis C infected liver, the disease is predictably destructive. For this reason, identification of patients at high risk for the development of cancer would allow for: 1) closer surveillance and 2) chemoprevention protocols. The major risk factors of HCC include infection with Hepatitis B or C virus (HBV or HCV), with the highest risk occurring when patients develop cirrhosis. It is estimated that patients with HCV and cirrhosis have much higher risk (15-20 fold) to develop HCC [5]. 

Serologic biomarkers such as alpha-fetoprotein (AFP) have been used to help diagnose or assess prognosis in HCC for decades. In patients with inflammatory conditions such as hepatitis, the value of AFP is limited as AFP levels can be elevated beyond the threshold in the absence of measureable cancer and negative in cases of obvious malignancy [6]. For this reason, the physician cannot argue for an intervention, such as liver transplant, based on AFP alone. This lack of specificity diminishes its value in screening hepatitis patients [6,7,8,9]. Other serum markers, such as serum *Lens culinaris* agglutinin-reactive AFP (AFP-L3), des γ-carboxy prothrombin (DCP) and the secreted isoforms of ERBB3 (sERBB3) have been observed to have better performance for the diagnosis of HCC [10,11,12,13,14]. However, most of these markers have not been integrated into clinical practice.

Given the importance of liver function in metabolism, metabolite biomarkers might provide alternative biomarker candidates. In particular, metabolite profiling provides a broad and systematic view of metabolic change in complex biological samples and can be potentially useful for identifying metabolite biomarkers. Utilizing high-throughput analytical techniques such as nuclear magnetic resonance spectroscopy (NMR) and mass spectrometry (MS), metabolite profiling provides a detailed and quantitative analysis of 10s to 100s of metabolites and has therefore been applied to numerous areas including drug response, early disease diagnosis, toxicity and nutritional studies. [15,16,17,18]. A number of biomarker candidates have been proposed for different cancers, including lung [19,20], prostate [21], colon [22], breast [23,24] and esophageal [25,26]. 

Several metabolite-profiling studies have focused on detecting HCC in different patient populations. Yang *et al.* applied high-resolution magic-angle spinning (HRMAS) in order to study adjacent, high-grade and adjacent low-grade liver cancer tissues and found several metabolites that clearly differentiated the samples, including lactate and several amino acids [27]. NMR was also used to screen urine samples from HCC patients in a Nigerian population [28]. Multivariate, partial least squares discriminant analysis (PLS-DA) models, based on markers such as creatinine, carnitine, creatine and acetone, were found to differentiate HCC patients from both healthy controls and patients with cirrhosis with high accuracy. The use of liquid chromatography (LC)-MS and gas chromatography (GC)-MS has also been made to discover promising metabolite marker candidates, including amino acids and lipids [29,30,31,32,33]. These studies have identified metabolites with high classification accuracy, revealing metabolite profiling to be a promising approach. However, additional studies are needed; specifically, studies focusing on metabolite markers that distinguish patients with a risk of developing HCC. Many of the earlier studies have focused on separating HCC patients and healthy controls, which is less relevant clinically since healthy subjects are unlikely to develop HCC. Second, several of the metabolite marker candidates were discovered based on a limited number of samples and lack sufficient validation. Additionally, only a few of these studies focus on the population of the U.S. Considering that the risk of HCC differs across regions and ethnic groups, studies on different populations are also important.

In the present work, serum samples from 40 HCC patients with underlying HCV were collected before radiation or chemotherapy treatments, and 22 HCV patients with cirrhosis were studied. Most of these patients are Caucasians. Metabolite profiles were performed using ^1^H NMR and analyzed statistically using several approaches including partial least squares discriminant analysis (PLS-DA). A good model could be built based on the entire NMR spectrum as well as on only three metabolite biomarkers, and these results were internally cross-validated. This study is the first to identify good serum metabolite biomarkers by NMR to distinguish HCC patients from a population of patients with HCV and cirrhosis in the U.S.

## 2. Experimental Methods

### 2.1. Chemicals

Deuterium oxide (D_2_O, 99.9% D) and sodium azide (NaN_3_) were purchased from Cambridge Isotope Laboratories, Inc. (Andover, MA). The sodium salt of trimethylsilylpropionic acid-d_4_ (TSP), used as the internal standard, was from Sigma-Aldrich (Milwaukee, WI). All chemical reagents were analytical grade and used without further purification.

### 2.2. Serum Sample Collection and Storage

Human serum samples (n = 62) were obtained from the Indiana University/Lilly tissue bank, and consisted of two cohorts: HCC patients (n = 40) with underlying HCV, and HCV patients (n = 22) without HCC. A summary of sample information can be seen in Table 1. Frozen samples were transported to Purdue University under dry ice and then kept at -80 °C until analysis. The study was approved by the Institutional Review Boards at both Purdue University and Indiana University School of Medicine.

**Table 1:** Summary of demographic and clinical information for subjects recruited for the study.

	HCC (Hepatocellular carcinoma)	HCV (Hepatitis C Virus)
Samples	40	22
Average Age	54.6 ± 9.8	52.2 ± 8.1
Gender (F/M)	0.21	0.46
***Ethnicity***		
Caucasian	32	20
African American	1	2
Hispanic	3	0
Unknown	4	0

### 2.3. Sample Preparation and Acquisition of NMR Spectra

Samples were prepared by mixing 400 µL serum with 5µL sodium azide (0.01% w/v) and 130 μL D_2_O. The solution (530 µL) was then transferred to a 5-mm NMR tube. A 60 μL, 0.5mM TSP solution contained in a capillary insert was used as an internal standard. For the 1D NMR experiments, the spectra were acquired at 298 K on a Bruker Avance-500 spectrometer equipped with a TXI gradient cryoprobe, using standard 1D NOESY and 1D CPMG (Carr-Purcell-Meiboom-Gill) pulse sequences, each coupled with water presaturation. For each spectrum, 128 transients were collected with 16k time domain data points and using a spectral width of 6,000 Hz. All spectra were Fourier transformed using a 1.0 Hz exponential line broadening. Each acquired spectrum was then phased, baseline corrected and aligned with reference to alanine (δ=1.479 ppm) using Bruker Topspin 3.0 software.

### 2.4. Statistical Analysis

After excluding the spectral region δ 4.7–5.2 ppm containing the residual water resonance, each spectrum was binned to 4096 points (bin size 0.003 ppm), and then normalized to the area of the TSP signal at 0.0 ppm. The spectral data from both the CPMG and NOESY experiments were initially mean centered and subjected to orthogonal-signal-corrected (OSC) partial least squares (PLS) analysis using Matlab (R2008a; Mathworks, Natick, MA) and the PLS Toolbox (version 4.11, Eigenvector Research Inc.). 

In a second, more targeted analysis, a total of 19 metabolites were identified in the CPMG spectra by comparing their chemical shifts and multiplicities with the Human Metabolome Data Base [34]. The individual spectral regions for each of the 19 metabolite signals were then integrated. After auto-scaling, these peak integrals for both the HCC patients (n = 40) and HCV patients (n = 22) were subjected to principal component analysis (PCA) as well as partial least squares discriminant analysis (PLS-DA) with 7-fold internal cross-validation for model building. A receiver operating characteristics (ROC) curve was used to evaluate the performance of the model. Monte Carlo Cross Validation (MCCV) with 200 iterations was used to assess the model robustness using Matlab, PLS Toolbox version 4.11 and a home-developed code. For each of the iterations, the whole dataset was randomly divided into the training set (60% of the whole data set) and a testing set (40%). A PLS-DA model was built on the training set with 7-fold internal cross-validation to predict the validation set. The internal cross-validation prediction on the training set and the external prediction of the validation set were combined as the predicting result for each MCCV run. The overall true positive and true negative numbers were summarized, after which the sensitivity and specificity were calculated and compared with the results of a permutation analysis. In the permutation, the sample classification was randomly permuted and 200 MCCV iterations were performed as above. 

Third, feature selection using the Student’s *t*-test was performed for each metabolite between the HCC and HCV cohorts to focus the analysis on the most important metabolites for classification. Three significant metabolites (valine, creatinine and choline) with low (uncorrected, *vide infra*) *p*-values (<0.05) were selected as potential biomarkers. A new PLS-DA model was built, followed by MCCV and permutation with 200 iterations. Except for using 3 metabolite signals instead of 19, all the other procedures are the same as above. PCA analysis was also performed on these 3 biomarkers.

## 3. Results

**Figure 1:**
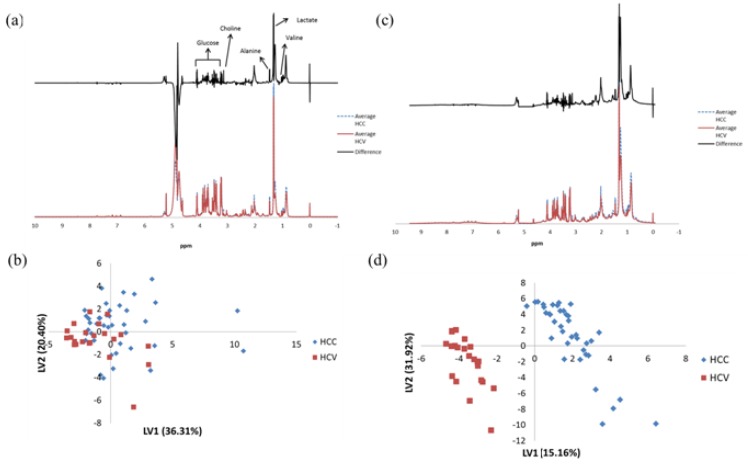
**(a)** The averaged Carr-Purcell-Meiboom-Gill (CPMG) spectra (bottom) for the HCC patients (blue dashed line, n=40) and HCV patients (red solid line n=22), along with the difference spectrum (top, black solid line). Major differences in metabolites are indicated in the difference spectrum. (**b**) Score plot for the OSC-PLS analysis of the ^1^H CPMG NMR spectra for all samples. (**c**) and (**d**) are the same as (**a**) and (**b**) except that they pertain to NOESY spectra.

The CPMG and NOESY spectra, averaged over the samples from each of the HCC and HCV patient cohorts, along with a difference spectrum, are shown in Figure 1 (a) and (c), respectively. We can observe clear changes in the CPMG spectra from several of the metabolite signals, including those from glucose, valine, alanine, lactate and choline. The changes from NOESY spectra are also clear, with most contributions coming from broad lipid signals. However, the large variation between samples makes it difficult to give any solid conclusion. The metabolic differences in both the NOESY and CPMG spectra between HCC and HCV patients can be identified using OSC-PLS analysis. The score plot for OSC-PLS analysis of the CPMG spectra is shown in Figure 1 (b). The two patient cohorts are separated and clustered in different areas of this score plot, with a few HCC samples overlapping the HCV region. The AUC for separation along LV1 was 0.71, with moderate sensitivity (0.74) but poor specificity (0.60). The loading plot (Supplemental Figure S1) indicates a number of peaks contribute to the separation. The score plot from the OSC-PLS analysis of the NOESY spectra shown in Figure 1 (d), shows an even better separation between the two patient cohorts, and the loading plot (Supplemental Figure S2) shows mostly lipid peaks. These results show promise for the future study of lipids. However, a major challenge in using NOESY to study lipids is that it cannot fully distinguish lipids with different fatty acid chains as they overlap. As a result, the following analysis will focus on CPMG spectra since they contain a larger number of peaks from identifiable and quantifiable metabolites.

Considering the contribution to the loading plots from many low-lying and unidentified metabolite peaks, as well as noise, a more targeted approach was also pursued. Individual peaks from 19 known metabolites (See Supplemental Information Table S1) were integrated and analyzed to reduce the contribution from chemical noise and to focus the analysis on known metabolite species so as to provide more mechanistic information. Initially, PCA analysis was performed on the 19 metabolites to see the data clustering. The results are shown in Figure S3; as anticipated, clear separation of the two groups was not observed in the PCA results. A PLS-DA model was built based on these metabolite signals to investigate classification and discrimination. The cross-validated prediction result and ROC curve are shown in Figure S4. The two sample classes are somewhat separated by this model, but a number of misclassifications still exist. The area under curve (AUC) is 0.71. 

The model was further tested by MCCV, and the results of the classification confusion matrix are shown in Supplementary Table S2. The low sensitivity (54%) and specificity (58%) that result from the MCCV procedure indicate that the model is not very strong. However, this model is still better than the permutation result (these data are provided in Table S2 as the values in parentheses). The sensitivity and specificity of the permutation test are only 50% and 48%, respectively, which is essentially a random result, as anticipated. The sensitivity and specificity results for both the true model and permutation test from 200 iterations are also plotted (see Supplemental Information Figure S5). Although not very impressive there is still some separation, which indicates that the predictive model is better than a random one. 

**Table 2:** Summary of three metabolites having low p-values.

Metabolite	Chemical Shift (ppm)	Multiplicity	p-value^a^	Fold change^b^
			(HCC *vs.* HCV)	
Choline	3.20	s	0.0200	1.32
Valine	1.03	d	5.67 × 10^−6^	1.53
Creatinine	3.03	s	0.0279	-1.28

Notes: a. *p*-values were calculated using the Student's unpaired *t*-test for peak integrals with local baseline correction and incorporating spectral normalization using TSP; b. A positive fold change indicates upregulation in HCC; while a negative fold change indicates upregulation in HCV.

**Figure 2:**
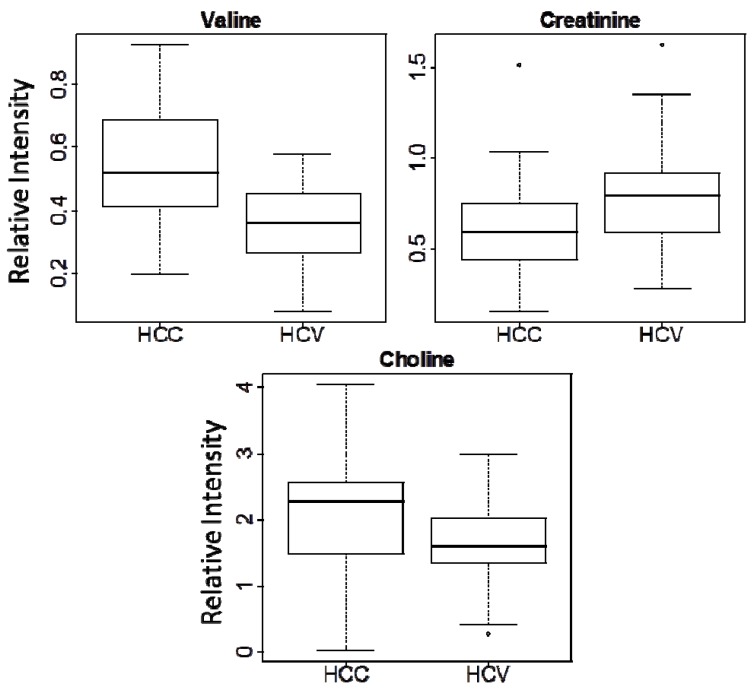
Box-plots for three metabolite markers in all the samples of this study (HCC *vs.* HCV).

Analysis of the PLS-DA loading plots (Supplemental Figure S6) indicated that only a few metabolites, such as valine, choline, alanine, creatine and asparagine, contributed to the separation. Feature selection was therefore used to further filter the metabolite signals and focus the analysis on the true differences between the two patient cohorts. *P*-values from the unpaired Student’s *t*-test were calculated for all 19 metabolites, and those metabolites with *p* < 0.05 were selected. Only three metabolites (choline, valine, and creatinine) passed this filter, and the *p*-values, fold changes, NMR chemical shifts and multiplicities for these three metabolites are listed in Table 2. Box-plots of the intensity data for the three metabolites (Figure 2) indicate that choline and valine are up-regulated in HCC, while creatinine is down-regulated.

**Figure 3:**
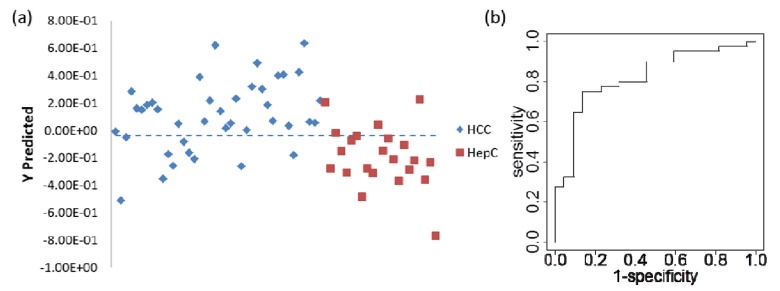
PLS-DA results for the model based on 3 potential metabolite biomarkers for differentiating HCC and HCV patient samples. **(a)** Cross-validation predicted class values. **(b)** Receiver operating characteristics (ROC) curve of the prediction result, with AUC of 0.83.

A new PLS-DA model was built based on the three metabolites, and the cross validation prediction results are shown in Figure 3. A much better result can be seen both in the classification and the ROC curve. The new AUC is 0.83, indicating that this is an improved model. A sensitivity of 80% can be obtained with a specificity of 71%, outperforming the clinical marker AFP, which has a sensitivity of 41% to 65% and specificity of 80% to 94% when using AFP level > 20 microg/L as the cutoff for HCC *vs.* HCV [35]. PCA analysis on these three markers showed some separation along PC1 as shown in Figure S7.

**Figure 4:**
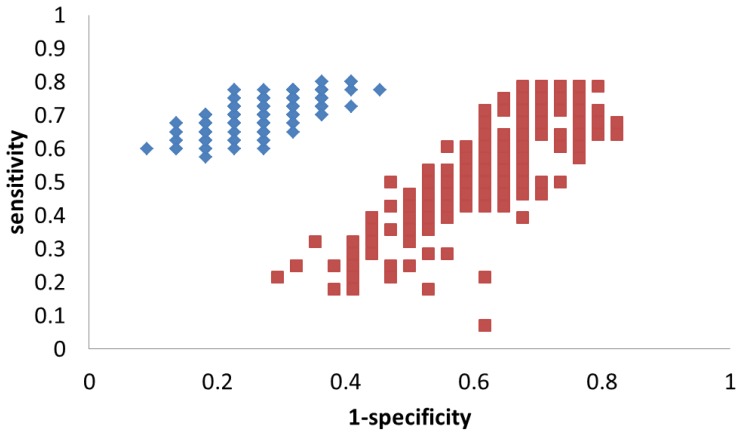
Results of the MCCV results (200 iterations) shown in ROC space for PLS-DA models based on the 3 metabolites used to discriminate HCC from HCV. Each blue diamond represents an iteration of the true model; each red square represents an iteration of the permutation model.

To better evaluate the robustness of this model, the same MCVV and permutation were used again, and the results can be found in Table 3. This time, the average sensitivity and specificity are 71% and 73% for the true model, a significant increase over the results of the model based on 19 metabolites. As expected, the permutation results show essentially a random distribution (sensitivity = 54% and specificity = 39%). To better visualize the difference, the results of the MCCV procedure are plotted in Figure 4. True model results cluster towards the top-left corner of the plot, representing good sensitivity and specificity. The permutation results are spread about the center of the plot and are well separated from the true model.

**Table 3:** Confusion matrix calculated from PLS-DA using 3 serum biomarkers for the HCC (n = 40) and HCV (n = 22) patients using 200 Monte-Carlo cross validation (MCCV) iterations. The numbers in parentheses are the results from permutation analysis.

		Predicted class	
True class	Total number of samples	HCC	HCV
HCC	8000 (8000)	5674 (4349)	2326 (3651)
HCV	4400 (4400)	1195 (2735)	3205 (1665)

## 4. Discussion

A metabolite profiling approach was applied to identify biomarker candidates for distinguishing HCC patients within an HCV population. The effectiveness of current HCC surveillance markers or methods such as alpha-fetoprotein (AFP) and abdominal ultrasound (US) are limited by low sensitivity and specificity. Hence the effectiveness of such approaches in reducing HCC mortality has remained modest [36]. Improved detection methods, such as blood-based biomarkers, are needed to improve this situation.

Metabolite biomarkers provide an opportunity to enhance the detection of HCC [29,33]. As shown in the present work, the entire ^1^H NMR spectrum can be used to develop a diagnostic metabolite profile with good sensitivity and specificity [20]. This approach is based on the combination of a large number of metabolite signals, many of which have not yet been identified. The use of feature selection, based on the Student’s *t*-test, resulted in 3 relatively strong biomarker candidates. We decided to use the uncorrected *p*-values, in part because each of these biomarker candidates has some precedence in cancer metabolism and due to our desire to avoid possible false negatives. In the case of creatinine, a gender imbalance in the two patient cohorts may be reducing its significance (*vide infra*). The resulting PLS-DA model based on these 3 metabolites shows good performance, at least better than the model based on the entire CPMG NMR spectrum. In contrast, the use of 19 metabolites without the use of feature selection performs much more poorly. The OSC-PLS analysis of the full NOESY spectra showed a clear separation between HCC and HCV patients, and this approach may be quite useful for distinguishing these cohorts. Future studies to validate these findings are planned, including an investigation of which types of lipids are contributing to the separation. However, the combination of isolated lipid signals and identified metabolites could not provide an improved model compared with the one built using the 3 metabolites (data not shown). 

An investigation of age and gender effects on the model was also performed to evaluate possible confounding effects. The averages and standard deviations of the age distributions in HCC and HCV groups are quite similar, indicating that there is no confounding effect to be anticipated due to age. However, the gender distribution differs significantly between the two groups. We, therefore, performed a Student’s *t*-test for the 3 markers between the male and female patients in each of the two patient cohorts. All *p*-values were above 0.05 (see Supplemental Table S3), indicating that any gender effect can be neglected for these metabolites in this study. The results also show that the disease effect on creatinine levels dominated any gender effect; creatinine increased overall, in males compared with females, and in females with HCV compared to those with HCC. Interestingly, the increase in creatinine levels for females was highly significant (Table S4). 

The three metabolites identified by feature selection do have some precedence as biomarkers. Creatinine was found to decrease in the samples from HCC patients compared to those from patients with HCV without cancer. Unique to this study was the ability to show differences within two diseased states, as opposed to other studies that focused on differences between diseased states (cirrhosis or cancer) compared to normal controls. For example, creatinine was seen to decrease in the urine of liver cancer patients compared with healthy controls as detected by MS [37]. In an NMR study focused on African subjects, creatinine was lower in urine samples of patients with cirrhosis compared to the urine from healthy controls [28]. More recently, creatinine was found to be decreased in the serum of patients with HCC compared with healthy subjects [33]. Corroborating its potential role as a cancer biomarker, aberrations in serum or urine creatinine levels were also associated with other cancers such as lung cancer (in urine) [20], pancreatic cancer (in serum) [38], esophageal cancer (in serum) [25] and colorectal cancer (in urine) [39]. Creatinine levels are generally higher in males than in females and correlate with muscle mass [40]. It is important to emphasize that studies with unmatched gender participation can result in biased results for metabolites that are sensitive to gender. However, in this study, we find that the HCC patient group, which does have a significantly larger number of males compared to the HCV group, actually exhibits a *lower* concentration of creatinine, indicating a definitive pathological role for creatinine. In fact, among female patients alone the creatinine change from HCV to HCC is quite significant (*p*=0.003, Supplemental Tables S3 & S4 and Figure S8). One can anticipate that better gender-matched cohorts might well increase the significance of creatinine as a biomarker for HCC. Nevertheless, the specific molecular mechanism of its association with HCC and/or HCV remains to be explored. 

In contrast, valine and choline were found to be upregulated in HCC patients. The elevation of valine has been observed in HCC tissue [27] and blood [41], as well as the serum of HBV infected cirrhosis patients [42]. An important step of valine catabolism occurs largely in the liver. This step involves oxidative decarboxylation of branched-chain α-keto acids generated from valine and other branched-chain amino acids in extrahepatic tissues [43,44]. Previous studies showed that methacrylyl-coenzyme A (MC-CoA), a toxic compound generated in valine catabolism, is less detoxified in HCC or cirrhosis patients. MC-CoA induces a change of valine metabolism resulting in increased serum valine [45]. It is worth noting that changes in valine levels have been found in some digestive system cancers, such as oral cancer [46] and gastric cancer [47]. 

Changes in choline metabolism have also been related with HCC previously. The Lin group found decreased choline in HCC and cirrhosis patient sera compared with normal sera, although they did not compare HCC and cirrhotic patients [48]. In HCC tissue, choline was found upregulated [27], which is consistent with previous *in vivo* MRS studies [49]. Generally, choline is an essential metabolite in the synthesis of phospholipids for cancer cell membranes [50]. This metabolism has been studied and monitored by NMR previously [51,52,53]. Choline is also associated with many cancer types. For example, it has shown to be associated with colorectal cancer [54], high grade gliomas [55], and breast cancer [56]. Thus, the metabolism of the membrane phospholipids caused by accelerated cell proliferation could be a reason for elevated choline in the sera of HCC patients [27].

## 5. Conclusions

^1^H NMR metabolic profiling of serum samples has been shown to differentiate HCC from HCV patients. In addition to a good separation based on broad lipid signals in the NMR spectra, three metabolites, creatinine, valine and choline, were found to differentiate the two disease groups and each metabolite has some precedence as a potential HCC biomarker in human serum or urine. In addition, these metabolites are readily detected in serum by a number of analytical methods, indicating that upon further validation they could be straightforwardly translated into clinical practice. 

A distinguishing feature of this study is that it focuses on a particularly challenging patient cohort, *i.e.*, those with underlying HCV. It is extremely difficult to differentiate HCC patients with underlying HCV from HCV patients for several reasons: 1) mediators associated with inflammation often overlap with those associated with cancer and therefore teasing out cancer specific differences is difficult; 2) changes associated with fibrosis also overlap with cancer and the majority of HCV patients do not develop cancer until the liver has become severely fibrotic; and 3) confirmation of cancer requires pathologic evidence that is not found in cases where resection or transplant has not been performed or where occult disease is present, but only detected from the most sophisticated tests. Patients with HCV were of particular interest for this study since they represent the largest cohort of HCC patients within the US and are at the highest risk for developing HCC during their lifetimes. The results of this study indicate the promise of developing metabolite profiles for the detection of HCC. Future studies will focus on adding MS detected biomarker candidates and expansion of the studies with additional sample cohorts. We anticipate that additional metabolite biomarkers will significantly improve the detection model and provide an alternative to current modalities.

## Supplementary Files

Supplementary File 1 PDF-Document (PDF, 762 KB)

## References

[1] Shariff M.I., Cox I.J., Gomaa A.I., Khan S.A., Gedroyc W., Taylor-Robinson S.D. (2009). Hepatocellular carcinoma: Current trends in worldwide epidemiology, risk factors, diagnosis and therapeutics. Expert Rev. Gastroenterol. Hepatol..

[2] El-Serag H.B. (2011). Current concepts hepatocellular carcinoma. N. Engl. J. Med..

[3] El-Serag H.B., Marrero J.A., Rudolph L., Reddy K.R. (2008). Diagnosis and treatment of hepatocellular carcinoma. Gastroenterology.

[4] Tang Z.Y., Yu Y.Q., Zhou X.D. (1984). An important approach to prolonging survival further after radical resection of AFP-positive hepatocellular-carcinoma. J. Exp. Clin. Cancer Res..

[5] Donato F., Tagger A., Gelatti U., Parrinello G., Boffetta P., Albertini A., Decarli A., Trevisi P., Ribero M.L., Martelli C. (2002). Alcohol and hepatocellular carcinoma: The effect of lifetime intake and hepatitis virus infections in men and women. Am. J. Epidemiol..

[6] Kew M.C., Purves L.R., Bersohn I. (1973). Serum alpha-fetoprotein levels in acute viral-hepatitis. Gut.

[7] Tong M.J., Elfarra N.S., Reikes A.R., Co R.L. (1995). Clinical outcomes after transfusion-associated hepatitis-C. N. Engl. J. Med..

[8] Bayati N., Silverman A.L., Gordon S.C. (1998). Serum alpha-fetoprotein levels and liver histology in patients with chronic hepatitis C. Am. J. Gastroenterol..

[9] Hu K.Q., Kyulo N.L., Lim N., Elhazin B., Hillebrand D.J., Bock T. (2004). Clinical significance of elevated alpha-fetoprotein (AFP) in patients with chronic hepatitis c, but not hepatocellular carcinoma. Am. J. Gastroenterol..

[10] Gomaa A.I., Khan S.A., Leen E.L.S., Waked I., Taylor-Robinson S.D. (2009). Diagnosis of hepatocellular carcinoma. World J. Gastroenterol..

[11] Marrero J.A., Feng Z.D., Wang Y.H., Nguyen M.H., Befeler A.S., Roberts L.R., Reddy K.R., Harnois D., Llovet J.M., Normolle D. (2009). Alpha-fetoprotein, des-gamma carboxyprothrombin, and lectin-bound alpha-fetoprotein in early hepatocellular carcinoma. Gastroenterology.

[12] Mita Y., Aoyagi Y., Yanagi M., Suda T., Suzuki Y., Asakura H. (1998). The usefulness of determining des-gamma-carboxy prothrombin by sensitive enzyme immunoassay in the early diagnosis of patients with hepatocellular carcinoma. Cancer.

[13] Hsieh S.Y., He J.R., Yu M.C., Lee W.C., Chen T.C., Lo S.J., Bera R., Sung C.M., Chiu C.T. (2011). Secreted RRBB3 isoforms are serum markers for early hepatoma in patients with chronic hepatitis and cirrhosis. J. Proteome Res..

[14] Kumada T., Toyoda H., Kiriyama S., Tanikawa M., Hisanaga Y., Kanamori A., Tada T., Tanaka J., Yoshizawa H. (2011). Predictive value of tumor markers for hepatocarcinogenesis in patients with hepatitis C virus. J. Gastroenterol..

[15] Kaddurah-Daouk R., Kristal B.S., Weinshilboum R.M. (2008). Metabolomics: A global biochemical approach to drug response and disease. Annu. Rev. Pharmacol..

[16] Gowda G.A.N., Zhang S.C., Gu H.W., Asiago V., Shanaiah N., Raftery D. (2008). Metabolomics-based methods for early disease diagnostics. Expert Rev. Mol. Diagn..

[17] Gibney M.J., Walsh M., Brennan L., Roche H.M., German B., van Ommen B. (2005). Metabolomics in human nutrition: Opportunities and challenges. Am. J. Clin. Nutr..

[18] Griffin J.L. (2003). Metabonomics: NMR spectroscopy and pattern recognition analysis of body fluids and tissues for characterisation of xenobiotic toxicity and disease diagnosis. Curr. Opin. Chem. Biol..

[19] Rocha C.M., Carrola J., Barros A.S., Gil A.M., Goodfellow B.J., Carreira I.M., Bernardo J., Gomes A., Sousa V., Carvalho L. (2011). Metabolic signatures of lung cancer in biofluids: Nmr-based metabonomics of blood plasma. J. Proteome Res..

[20] Carrola J., Rocha C.M., Barros A.S., Gil A.M., Goodfellow B.J., Carreira I.M., Bernardo J., Gomes A., Sousa V., Carvalho L. (2011). Metabolic signatures of lung cancer in biofluids: Nmr-based metabonomics of urine. J. Proteome Res..

[21] Sreekumar A., Poisson L.M., Rajendiran T.M., Khan A.P., Cao Q., Yu J., Laxman B., Mehra R., Lonigro R.J., Li Y. (2009). Metabolomic profiles delineate potential role for sarcosine in prostate cancer progression. Nature.

[22] Chan E.C.Y., Koh P.K., Mal M., Cheah P.Y., Eu K.W., Backshall A., Cavill R., Nicholson J.K., Keun H.C. (2009). Metabolic profiling of human colorectal cancer using high-resolution magic angle spinning nuclear magnetic resonance (HR-MAS NMR) spectroscopy and gas chromatography mass spectrometry (GS/MS). J. Proteome Res..

[23] Slupsky C.M., Steed H., Wells T.H., Dabbs K., Schepansky A., Capstick V., Faught W., Sawyer M.B. (2010). Urine metabolite analysis offers potential early diagnosis of ovarian and breast cancers. Clin. Cancer Res..

[24] Asiago V.M., Alvarado L.Z., Shanaiah N., Gowda G.A.N., Owusu-Sarfo K., Ballas R.A., Raftery D. (2010). Early detection of recurrent breast cancer using metabolite profiling. Cancer Res..

[25] Zhang J., Liu L.., Wei S., Gowda G.A.N., Hammoud Z., Kesler K.A., Raftery D. (2011). Metabolomics study of esophageal adenocarcinoma. J. Thorac. Cardiovasc. Surg..

[26] Zhang J., Bowers J., Liu L., Wei S., Gowda G.A.N., Hammoud Z., Raftery D. (2012). Esophageal cancer metabolite biomarkers detected by LC-MS and NMR methods. PloS One.

[27] Yang Y.X., Li C.L., Nie X., Feng X.S., Chen W.X., Yue Y., Tang H.R., Deng F. (2007). Metabonomic studies of human hepatocellular carcinoma using high-resolution magic-angle spinning 1H NMR spectroscopy in conjunction with multivariate data analysis. J. Proteome Res..

[28] Shariff M.I.F., Ladep N.G., Cox I.J., Williams H.R.T., Okeke E., Malu A., Thillainayagam A.V., Crossey M.M.E., Khan S.A., Thomas H.C. (2010). Characterization of urinary biomarkers of hepatocellular carcinoma using magnetic resonance spectroscopy in a Nigerian population. J. Proteome Res..

[29] Yang J., Xu G.W., Zheng Y.F., Kong H.W., Pang T., Lv S., Yang Q. (2004). Diagnosis of liver cancer using hplc-based metabonomics avoiding false-positive result from hepatitis and hepatocirrhosis diseases. J. Chromatogr. B.

[30] Wu H., Xue R.Y., Dong L., Liu T.T., Deng C.H., Zeng H.Z., Shen X.Z. (2009). Metabolomic profiling of human urine in hepatocellular carcinoma patients using gas chromatography/mass spectrometry. Anal. Chim. Acta.

[31] Chen F., Xue J.H., Zhou L.F., Wu S.S., Chen Z. (2011). Identification of serum biomarkers of hepatocarcinoma through liquid chromatography/mass spectrometry-based metabonomic method. Anal. Bioanal. Chem..

[32] Patterson A.D., Maurhofer O., Beyoglu D., Lanz C., Krausz K.W., Pabst T., Gonzalez F.J., Dufour J.F., Idle J.R. (2011). Aberrant lipid metabolism in hepatocellular carcinoma revealed by plasma metabolomics and lipid profiling. Cancer Res..

[33] Chen T.L., Xie G.X., Wang X.Y., Fan J., Qui Y.P., Zheng X.J., Qi X., Cao Y., Su M.M., Wang X.Y. (2011). Serum and urine metabolite profiling reveals potential biomarkers of human hepatocellular carcinoma. Mol. Cell. Proteomics.

[34] Human Metabolome Database www.hmdb.ca.

[35] Gupta S., Bent S., Kohlwes J. (2003). Test characteristics of alpha-fetoprotein for detecting hepatocellular carcinoma in patients with hepatitis C—A systematic review and critical analysis. Ann. Intern. Med..

[36] El-Serag H.B., Kramer J.R., Chen G.J., Duan Z.G., Richardson P.A., Davila J.A. (2011). Effectiveness of afp and ultrasound tests on hepatocellular carcinoma mortality in hcv-infected patients in the USA. Gut.

[37] Chen J., Wang W.Z., Lv S., Yin P.Y., Zhao X.J., Lu X., Zhang F.X., Xu G.W. (2009). Metabonomics study of liver cancer based on ultra performance liquid chromatography coupled to mass spectrometry with hilic and rplc separations. Anal. Chim. Acta.

[38] OuYang D., Xu J.J., Huang H.G., Chen Z. (2011). Metabolomic profiling of serum from human pancreatic cancer patients using 1H NMR spectroscopy and principal component analysis. Appl. Biochem. Biotechnol..

[39] Cheng Y., Xie G., Chen T., Qiu Y., Zou X., Zheng M., Tan B., Feng B., Dong T., He P. (2012). Distinct urinary metabolic profile of human colorectal cancer. J. Proteome Res..

[40] Hultman E., Soderlund K., Timmons J.A., Cederblad G., Greenhaff P.L. (1996). Muscle creatine loading in men. J. Appl. Physiol..

[41] Xue R.Y., Lin Z.X., Deng C.H., Dong L., Liu T.T., Wang J.Y., Shen X.Z. (2008). A serum metabolomic investigation on hepatocellular carcinoma patients by chemical derivatization followed by gas chromatography/mass spectrometry. Rapid Commun. Mass Spectrom..

[42] Xue R.Y., Dong L., Wu H., Liu T.T., Wang J.Y., Shen X.Z. (2009). Gas chromatography/mass spectrometry screening of serum metabolomic biomarkers in hepatitis B virus infected cirrhosis patients. Clin. Chem. Lab. Med..

[43] Taniguchi K., Nonami T., Nakao A., Harada A., Kurokawa T., Sugiyama S., Fujitsuka N., Shimomura Y., Hutson S.M., Harris R.A. (1996). The valine catabolic pathway in human liver: Effect of cirrhosis on enzyme activities. Hepatology.

[44] Taniguchi K., Nonami T., Takagi H., Shimomura Y., Fujitsuka N., Harris R.A. (1995). Enzyme-activity in the valine catabolic pathway of human liver. Faseb J..

[45] Ishigure K., Shimomura Y., Murakami T., Kaneko T., Takeda S., Inoue S., Nomoto S., Koshikawa K., Nonami T., Nakao A. (2001). Human liver disease decreases methacrylyl-coa hydratase and beta-hydroxyisobutyryl-coa hydrolase activities in valine catabolism. Clin. Chim. Acta.

[46] Tiziani S., Lopes V., Gunther U.L. (2009). Early stage diagnosis of oral cancer using ^1^H NMR-based metabolomics. Neoplasia.

[47] Wu H., Xue R.Y., Tang Z.Q., Deng C.H., Liu T.T., Zeng H.Z., Sun Y.H., Shen X.Z. (2010). Metabolomic investigation of gastric cancer tissue using gas chromatography/mass spectrometry. Anal. Bioanal. Chem..

[48] Gao H.C., Lu Q., Liu X., Cong H., Zhao L.C., Wang H.M., Lin D.H. (2009). Application of ^1^H NMR-based metabonomics in the study of metabolic profiling of human hepatocellular carcinoma and liver cirrhosis. Cancer Sci..

[49] Kuo Y.T., Li C.W., Chen C.Y., Jao J.C., Wu D.K., Liu G.C. (2004). *In vivo* proton magnetic resonance spectroscopy of large focal hepatic lesions and metabolite change of hepatocellular carcinoma before and after transcatheter arterial chemoembolization using 3.0-T MR scanner. J. Magn. Reson. Imaging.

[50] Michel V., Yuan Z.F., Ramsubir S., Bakovic M. (2006). Choline transport for phospholipid synthesis. Exp. Biol. Med..

[51] Daly P.F., Lyon R.C., Faustino P.J., Cohen J.S. (1987). Phospholipid metabolism in cancer cells monitored by 31p nmr spectroscopy. J. Biol. Chem..

[52] Miller B.L. (1991). A review of chemical issues in 1H NMR spectroscopy: N-acetyl-l-aspartate, creatine and choline. NMR Biomed..

[53] Kuesel A.C., Graschew G., Hull W.E., Lorenz W., Thielmann H.W. (1990). 31P NMR studies of cultured human tumor cells. Influence of pH on phospholipid metabolite levels and the detection of cytidine 5'-diphosphate choline. NMR Biomed..

[54] Chen W.X., Zhou X.Y., Huang D., Chen F., Du X. (2011). Metabolic profiling of human colorectal cancer using high resolution 1H nuclear magnetic resonance spectroscopy. Chin. J. Chem..

[55] Bianchi L., De Micheli E., Bricolo A., Ballini C., Fattori M., Venturi C., Pedata F., Tipton K.F., Della Corte L. (2004). Extracellular levels of amino acids and choline in human high grade gliomas: An intraoperative microdialysis study. Neurochem. Res..

[56] Katz-Brull R., Seger D., Rivenson-Segal D., Rushkin E., Degani H. (2002). Metabolic markers of breast cancer: Enhanced choline metabolism and reduced choline-ether-phospholipid synthesis. Cancer Res..

