# Supplementary material for: Differentiating Hepatocellular Carcinoma from Hepatitis C Using Metabolite Profiling
Source: Metabolites. 2012 Oct 10;2(4):701–16. doi: 10.3390/metabo2040701 (PMC3901236; doi:10.3390/metabo2040701)
Supplement: Supplementary File 1 — PDF-Document (PDF, 762 KB) [file metabolites-02-00701-s001.pdf]

Supplemental Information

Figure S1. Loadings plot along LV1 for OSC-PLS analysis of the whole CPMG spectral data.

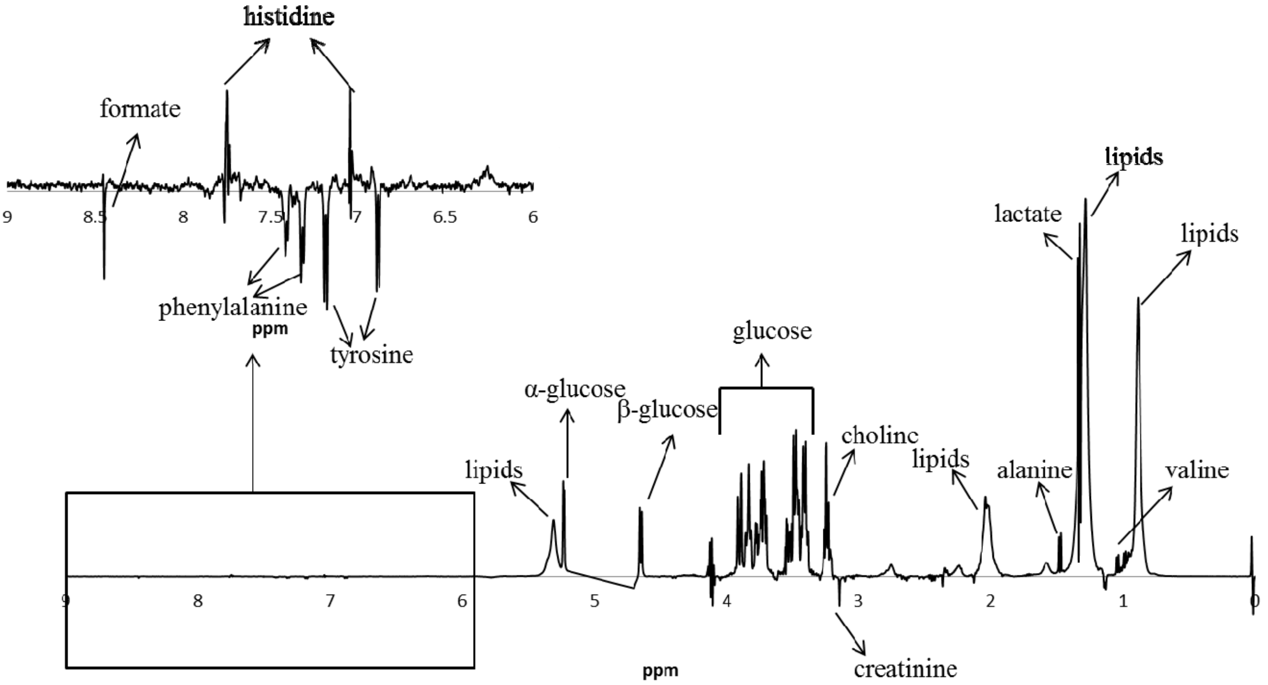

Figure S2. Loadings plot along LV1 for OSC-PLS analysis of the whole NOESY spectral data.

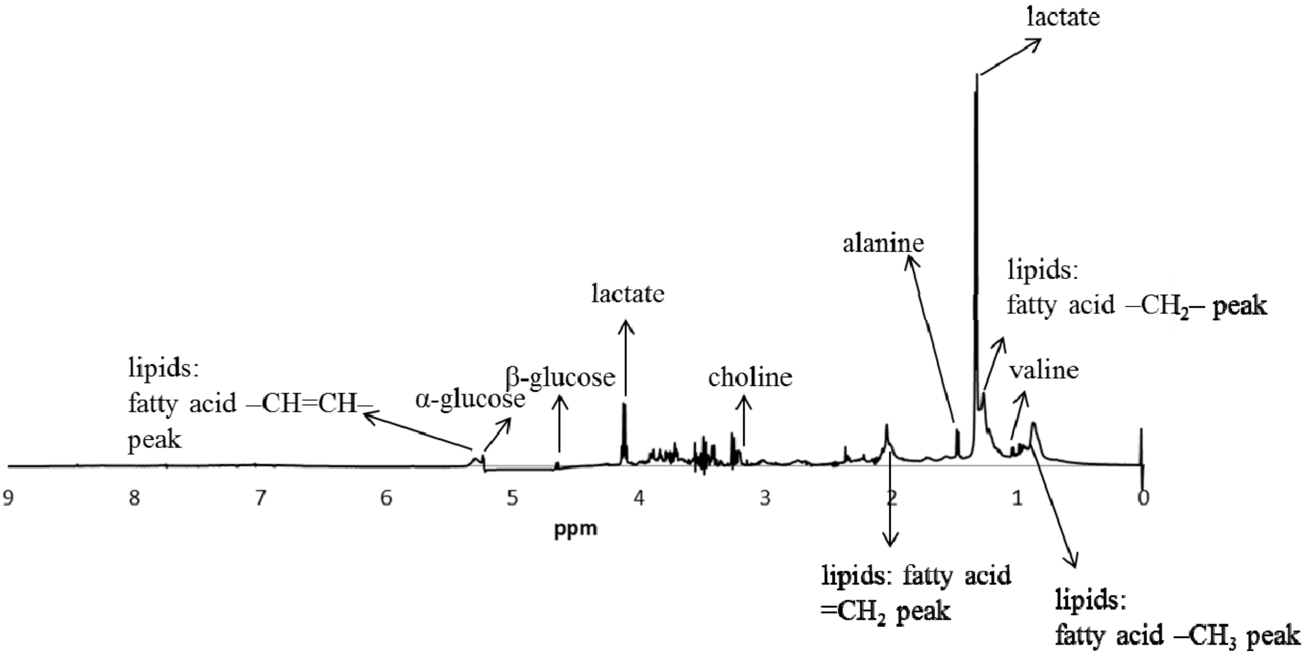

Figure S3. PCA analysis based on 19 identified metabolites in the spectra of the HCC and HCV samples. (A) Score plot. (B) Loading on PC2.

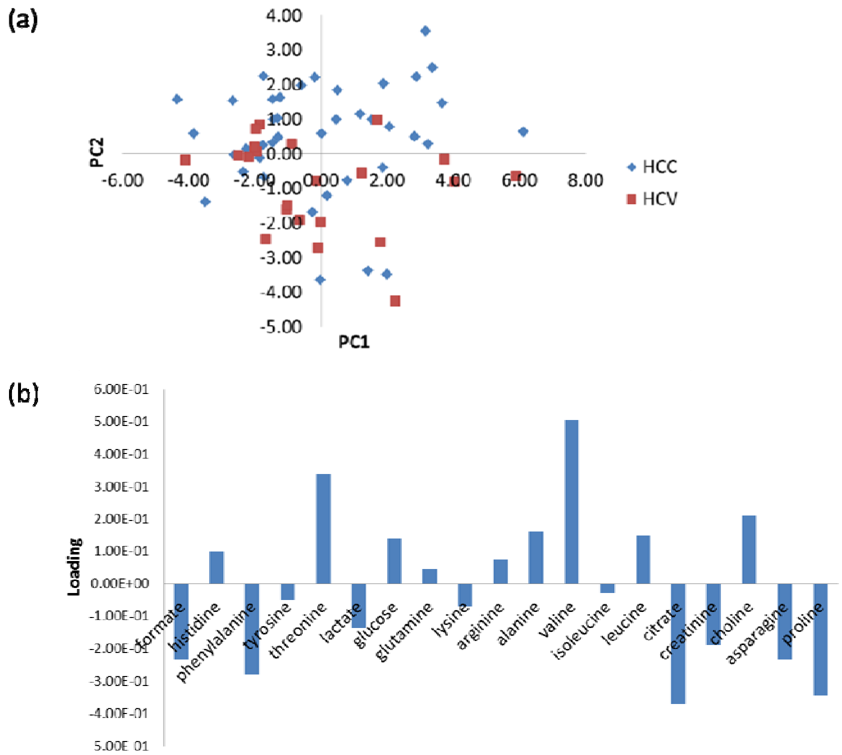

Figure S4. PLS-DA model based on 19 identified metabolites in the spectra of the HCC and HCV samples. (A) Cross-validation predicted class values. (B) ROC curve for the prediction result, with AUC of 0.71.

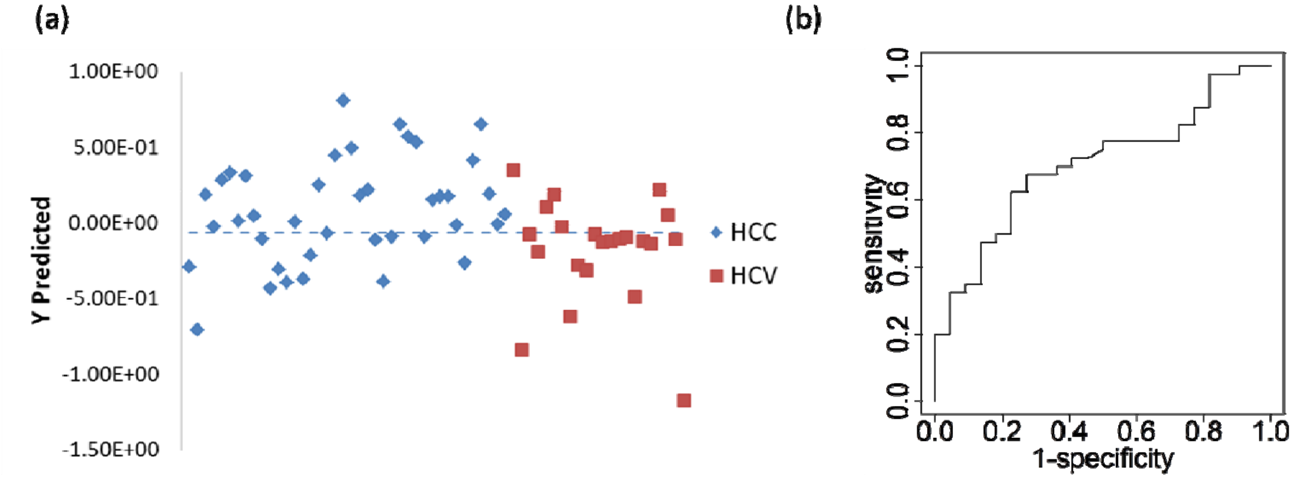

Figure S5. Results of the MCCV results (200 iterations) shown in ROC space for PLS-DA models based on the 19 metabolites used to discriminate HCC from HCV. Each blue diamond represents an iteration of the true model; each red square represents an iteration of the permutation model.

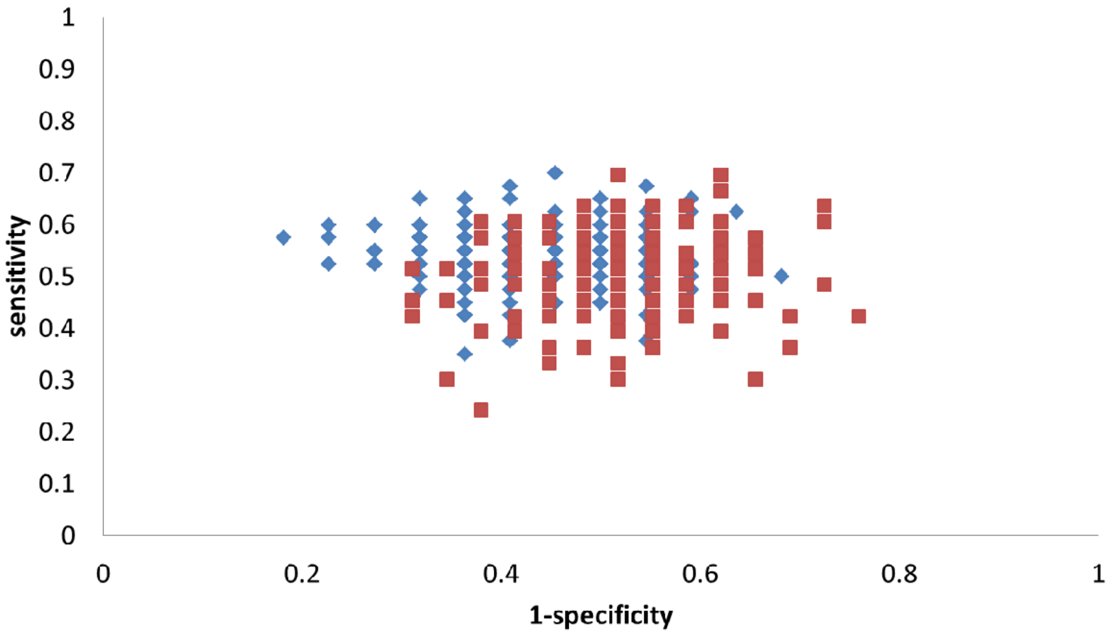

Figure S6. Plot of loadings of 19 metabolites for PLS-DA.

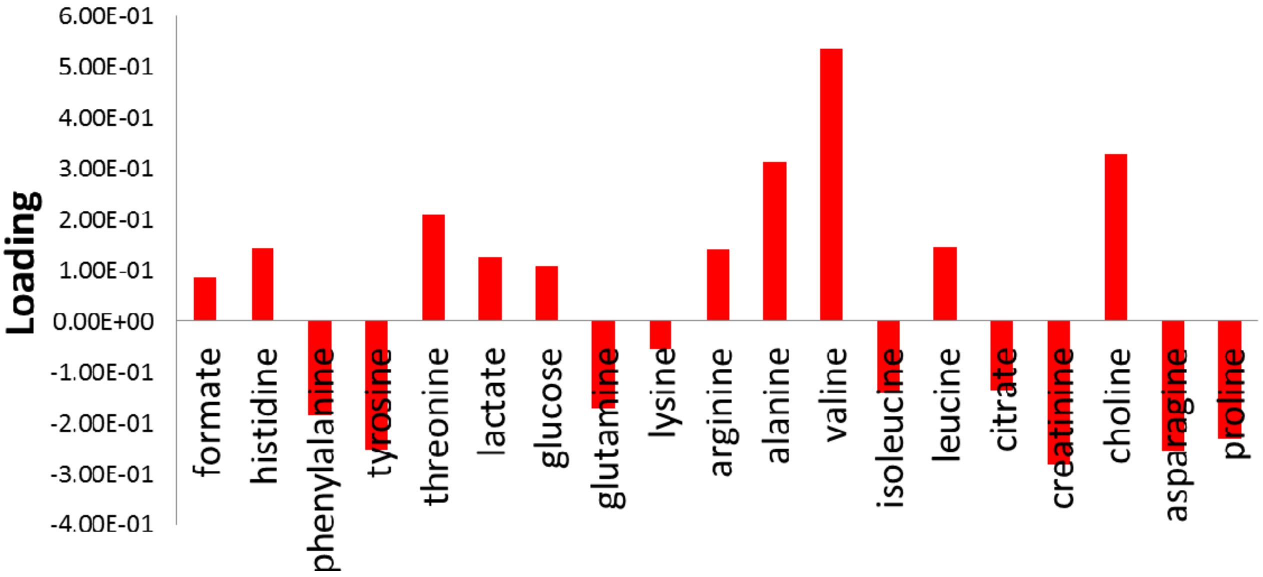

Figure S7. PCA analysis based on 3 potential biomarkers in the spectra of the HCC and HCV samples. (A) Score plot. (B) Loading on PC1.

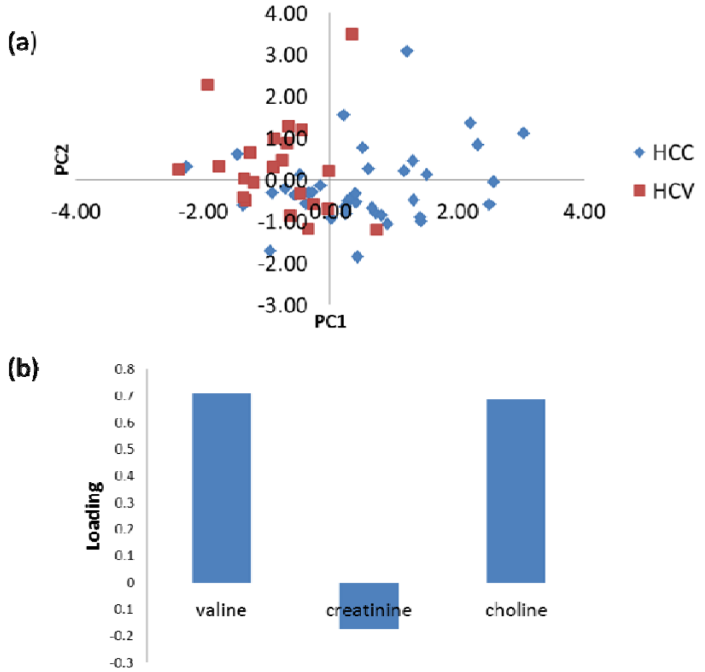

Figure S8. Box-plots for creatinine in the HCC and HCV groups for both male and female patients.

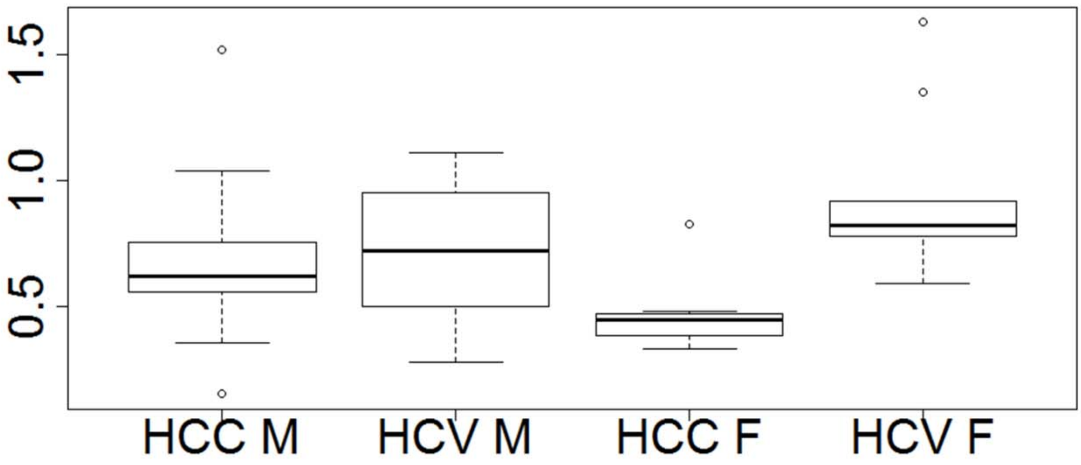

Table S1. Assignments of 19 metabolites.

Metabolite	Chemical shift (ppm)	multiplicity
**formate**	8.44	s
**histidine**	7.05	d
**phenylalanine**	7.36	m
**tyrosine**	6.94	m
**threonine**	4.25	m
**lactate**	4.1	q
**glucose**	5.23	d
**glutamine**	2.45	m
**lysine**	1.89	m
**arginine**	1.68	m
**alanine**	1.48	d
**valine**	1.03	d
**isoleucine**	0.92	t
**leucine**	0.94	t
**citrate**	2.53	d
**creatinine**	3.03	s
**choline**	3.2	d
**asparagine**	2.94	m
**proline**	4.08	dd

Table S2. Confusion matrix calculated from PLS-DA using 19 serum metabolites for the HCC (n=40) and HCV (n=22) patients using 200 MCCV iterations. The numbers in parentheses are the results from permutation analysis.

True class	Total number of samples	Predicted class	
		HCC	HCV
HCC	8000 (8000)	4345 (4027)	3565 (3973)
HCV	4400 (4400)	1865 (2280)	2535 (2120)

Table S3. P-values of male vs female in the two different sample cohorts for the three biomarkers.

Metabolite	p-value in HCC (Male vs Female)	p-value in HCV (Male vs Female)
Choline	0.53	0.41
Valine	0.55	0.06
Creatinine	0.06	0.12

Table S4. P-values for HCC vs HCV in both male and female patient populations for three biomarkers.

Metabolite	p-value in male (HCC vs HCV)	p-value in female (HCC vs HCV)
Choline	0.03	0.05
Valine	0.002	0.0007
Creatinine	0.5	0.003
